# Phased therapeutic intervention improves long-term prognosis of new hemodialysis patients

**DOI:** 10.12669/pjms.40.11.9405

**Published:** 2024-12

**Authors:** Jiakang Sun, Fei Guo, Deheng Wan, Yan Wang

**Affiliations:** 1 Jiakang Sun, Department of Nephrology, Qinhuangdao Haigang Hospital, Qinhuangdao 066000, Hebei, China; 2 Fei Guo, Department of Nephrology, Qinhuangdao Haigang Hospital, Qinhuangdao 066000, Hebei, China; 3 Deheng Wan, Department of Nephrology, Qinhuangdao Haigang Hospital, Qinhuangdao 066000, Hebei, China; 4 Yan Wang, Department of Nephrology, Qinhuangdao Haigang Hospital, Qinhuangdao 066000, Hebei, China

**Keywords:** Hemodialysis, Phased therapeutic intervention, Prognosis

## Abstract

**Objective:**

To explore the effect of phased therapeutic intervention in improving the long-term prognosis of new hemodialysis(HD) patients.

**Method:**

This was a retrospective study. A total of 90 new HD patients in Qinhuangdao Haigang Hospital from June 2021 to June 2022 were included and stratified according to their compliance. They were grouped using a random number generator. The control group was given routine nursing intervention, while the intervention group was intervened with phased change nursing intervention. The effects of different intervention modes on patient compliance, biochemical indicators, cardiac function indicators and inflammatory cytokines were compared.

**Results:**

At T_0_, the compliance of the two groups was improved compared with that before the intervention. The compliance of the intervention group at T_1_ and T_2_ (71.11%, 91.11%) was higher than that of the control group(48.89%, 75.56%), with statistically significant differences(*p<* 0.05). At T_0_, the levels of hemoglobin(Hb), hematocrit(Hct), serum iron(SI), transferrin saturation (TS) and left ventricular ejection fraction(LVEF) all increased compared with those before intervention(*p<* 0.05), At T_1_ and T_2_, Hb, Hct, SI, TS and LVEF in the intervention group were higher than those in the control group(*p<* 0.05). At T_1_ and T_2_, LVEDD, LVESD and LVWT in the intervention group were smaller than those in the control group(*p<* 0.05).

**Conclusion:**

Phased therapeutic intervention can significantly enhance the compliance of new HD patients, as well as further improve their anemia and cardiac function, and reduce inflammatory responses. Therefore, it is worthy of clinical application.

## INTRODUCTION

Hemodialysis(HD) is the main treatment for patients with end-stage renal disease (ESRD). The Chinese National Renal Data System (CNRDS) shows that the number of HD patients in China had reached about 560,000 by the end of 2017.^1^ HD, also known as blood purification, is the process which withdrawing blood from the body, fully mixing dialysate and blood for substance exchange in the dialyzer to eliminate metabolic wastes and excess water, and correct pH and electrolyte imbalance in the body, and then transfusing blood back into the body.^2^,^3^ Clinical practice has found that with the extension of dialysis time, the incidences of complications such as cardiovascular diseases, infections and malnutrition in HD patients increase gradually, which seriously affects their quality of life, resulting in high mortality. Their five year survival rate is only about 40%, which is lower than that of many cancers.^4^-^6^ It has been reported^7^,^8^ that the effect of HD treatment largely depends on patient compliance. However, new HD patients have poor compliance due to the lack of awareness of their own diseases and the high costs of long-term or even lifelong dialysis treatment, which is not conducive to their prognosis. Phased change intervention mode is a behavior intervention mode with phased changes as the main idea to implement phased nursing intervention according to the needs of patients in different behavioral phases, which can effectively improve the health behaviors of patients, and has good application effects in diabetes, lung cancer and cardio-cerebrovascular diseases.^9^ Based on this, the present study aimed to explore the effect of phased therapeutic intervention in improving the long-term prognosis of new HD patients.

## METHODS

In this was a retrospective study, 90 new hemodialysis (HD) patients in Qinhuangdao Haigang Hospital from June 2021 to June 2022 were divided into control group(n= 45) and intervention group(n= 45) according to different intervention modes.

### Ethical approval:

The study was approved by the Institutional Ethics Committee of Qinhuangdao Haigang Hospital (No.: 20211119; date: November 19, 2021), and written informed consent was obtained from all participants.

### Inclusion criteria:

- Aged 18~62 years.
- Initial diagnosis as ESRD, and maintenance hemodialysis (MHD) expected for more than 12 months.
- Ability to fill in relevant questionnaires independently.
- Informed research process and signed informed consent.

### Exclusion criteria:

- Accompanied by severe organic lesions of the heart, liver, kidney and other functional organs.
- History of malignant tumor, cardiac, cerebral or somatic surgery.
- Pregnant or lactating women.

In the control group, there were 25 males and 20 females, aged 18-62 years (average age, 48.25 ± 6.25 years). The intervention group included 21 males and 24 females, with an age of 20-67 years (average age, 48.50 ± 6.34 years). The baseline data showed no significant differences between the two groups(*p>* 0.05).

Control group Routine nursing intervention was performed during HD, including disease-related knowledge introduction, dietary guidance, dialysis guidance and nursing.

### Intervention group Phased change nursing intervention mode was used during HD:

***Pre-though + though phase:*** Through group education and one-to-one education, the potential anxiety and depression of the patients were relieved. Scientific and reasonable rehabilitation goals and plans were established based on their actual conditions. Their family problems at this phase were understood actively and solved as much as possible. Group education

***Preparation phase:*** The patients were provided with dietary guidance and material assistance, including explaining the dietary plan and precautions in detail, and listing the dietary list. They were asked to eat foods rich in high-quality proteins on the dietary list, such as lean meat, eggs, beans, etc. Small measuring spoons and graduated kettles were distributed, and salt- and water-intake control was instructed. At this phase, group education (45 min/time) and one-to-one education (30 min/time) were also carried out two times/week.

***Behavioral phase:*** Health education intervention was implemented mainly in the form of one-on-one education, 30 min/time, two times/week, including the pathogenesis of disease, methods for HD treatment, and the necessity of adhering to HD, so as to ensure that patients have a comprehensive understanding of their own conditions.

***Maintenance phase:*** The medical staff provided supervision and inspection, and held patient-staff meetings 30 min/time, one time a week. In the meetings, in addition to distributing health knowledge manuals, the patients were also invited and encouraged to speak actively, so as to help patients enhance their interpersonal skills.

### Observation indicators:

- ***Patient compliance***: Referring to the Development of Medical Treatment Adherence Scale for the End-stage Renal Disease Patients with Maintenance Hemodialysis:,^10^ the relative weight gain during HD (weight gain/dry weight during HD) and dietary compliance (serum potassium and phosphorus levels) were evaluated at the beginning (T_0_), 6 months (T_1_) and 12 months of HD (T_2_), respectively. Weight gain during HD = weight before this dialysis - weight after the last dialysis. Dry weight = ideal weight expected after HD (stable blood pressure, no edema or dehydration). Relative weight gain during HD > 5% was defined as non-compliance, and serum potassium > 5.5 mmol/L and serum phosphorus > 2.0 mmol/L as non-compliance. The patients with any above item were defined as non-compliant.
- ***Biochemical indicators:*** The levels of hemoglobin (Hb), hematocrit (Hct), serum iron (SI), transferrin saturation (TS), total iron binding capacity (TIBC), and serum ferritin (SF) in the peripheral blood/serum were detected using a fully automatic biochemical analyzer (Shanghai Jumu Medical Instruments Co., Ltd.) at T_0_, T_1_ and T_2_, respectively.
- ***Cardiac function:*** Left ventricular ejection fraction (LVEF), left ventricular end-diastolic diameter (LVEDD), left ventricular end-systolic diameter (LVESD), mean arterial pressure (MAP) and left ventricular wall thickness (LVWT) were measured by echocardiography (Shanghai Madison Medical Instruments Co., Ltd.), and the level of serum N-terminal pro-B-type natriuretic peptide (NT-proBNP) was quantitatively detected by electrochemiluminescence immunoassay at T_0_, T_1_ and T_2_, respectively.
- ***Inflammatory cytokines:*** The levels of serum interleukin-6 (IL-6), interleukin-8 (IL-8) and C-reactive protein (CRP) were detected at T_0_, T_1_ and T_2_, respectively, using enzyme-linked immunosorbent assay. The kits were purchased from Shanghai Mlbio Co., Ltd.

### Statistical analysis:

The data were statistically analyzed using SPSS 24.0, with a significant level at α = 0.05. The confidence interval was 95%, the measurement data conforming to the normal distribution were expressed as (*χ̅*±*S*), and analyzed by the LSD-*t* test. The counting data were expressed as (*n*, %), and analyzed with the *χ^2^* test.

## RESULTS

At T_0_, the compliance of the two groups was improved compared with that before the intervention. The compliance of the intervention group at T_1_ and T_2_ (71.11%, 91.11%) was higher than that of the control group (48.89%, 75.56%), with statistically significant differences (*p<*0.05). Table-I.

**Table-I:** Comparison of patient compliance between the two groups (*n*, %)

Group	T_0_	T_1_	T_2_
Control group (*n* = 45)	10(22.22)	22(48.89)^a^	34(75.56)^ab^
Intervention group (*n* = 45)	9(20.00)	32(71.11)^a^	41(91.11)^ab^
*χ^2^*	0.067	4.630	3.920
*P*	0.796	0.031	0.048

***Notes:*** Compared in the same group at T_0_, ^a^p< 0.05; compared in the same group at T_1_, ^b^p< 0.05.

At T_0_, the levels of Hb, Hct, SI and TS all increased, while TIBC and SF levels reduced in the two groups compared with those before intervention (*p<*0.05). At T_1_ and T_2_, the Hb, Hct, SI and TS levels were higher (*p<*0.05), while the TIBC and SF levels were lower (*p<*0.05) in the intervention group than those in the control group. Table-II.

**Table-II:** Comparison of biochemical indicators between the two groups (*χ̅*±*S*).

Group	Hb (g/L)			Hct (%)			SI (μmol/L)		

	T_0_	T_1_	T_2_	T_0_	T_1_	T_2_	T_0_	T_1_	T_2_
Control group (*n* = 45)	76.44±10.24	83.25±7.20^a^	92.25±6.58^ab^	24.16±4.10	26.44±3.95^a^	29.32±3.54^ab^	12.02±2.12	13.33±2.37^a^	14.67±3.67^ab^
Intervention group (*n* = 45)	76.80±10.15	89.25±7.50^a^	99.60±5.12^ab^	24.40±4.13	30.25±3.15^a^	36.25±2.54^ab^	12.19±2.05	14.80±3.05^a^	17.24±1.82^ab^
*t*	0.167	3.871	5.914	0.277	5.059	10.670	0.387	2.553	4.208
*P*	0.867	0.000	0.000	0.783	0.000	0.000	0.700	0.012	0.000

***Group***	***TS (%)***			***TIBC (μmol/L)***			***SF (μg/L)***		

	***T_0_***	***T_1_***	***T_2_***	***T_0_***	***T_1_***	***T_2_***	***T_0_***	***T_1_***	***T_2_***

Control group (*n* = 45)	10.70±2.19	15.64±2.97^a^	19.25±4.25^ab^	105.26±20.15	89.62±12.34^a^	75.62±8.01^ab^	365.25±52.61	245.25±31.20^a^	190.25±17.46^ab^
Intervention group (*n* = 45)	11.02±2.10	19.50±4.16^a^	27.62±5.01^ab^	106.32±20.37	80.62±10.07^a^	67.25±6.25^ab^	366.75±50.95	198.25±22.50^a^	160.25±12.25^ab^
*t*	0.707	5.066	8.546	0.248	3.791	5.526	0.137	8.196	9.435
*P*	0.481	0.000	0.000	0.805	0.000	0.000	0.891	0.000	0.000

***Notes:*** Compared in the same group at T_0_,

a p<0.05; compared in the same group at T_1_, ^b^p< 0.05.

At T_0_, LVEF in the two groups both increased compared with that before intervention (*p<*0.05). In the intervention group, LVEF was higher than that in the control group (*p<*0.05), LVEDD, LVESD and LVWT reduced than those before intervention (*p<*0.05), and MAP and NT-proBNP decreased than those before intervention (*p<*0.05) at T_1_ and T_2_. LVEDD, LVESD and LVWT were smaller (*p<*0.05), and MAP and NT-proBNP were lower in the intervention group compared with those in the control group at T_1_ and T_2_ (*p<*0.05). Table-III.

**Table-III:** Comparison of cardiac function indicators between the two groups (*χ̅*±*S*).

Group	LVEF (%)			LVEDD (mm)			LVESD (mm)		

	T_0_	T_1_	T_2_	T_0_	T_1_	T_2_	T_0_	T_1_	T_2_
Control group (n = 45)	52.61±4.26	55.26±3.90^a^	57.62±3.02^ab^	52.24±1.52	50.25±1.13^a^	46.90±3.85^ab^	40.21±1.02	38.57±1.08^a^	37.25±1.10^ab^
Intervention group (n = 45)	52.73±4.19	58.62±3.10^a^	61.20±2.52^ab^	52.39±1.46	48.25±2.82^a^	43.25±3.62^ab^	40.03±0.95	36.54±1.11^a^	34.90±1.25^ab^
*t*	0.135	4.524	6.106	0.477	4.416	4.633	0.866	8.793	9.468
*P*	0.893	0.000	0.000	0.634	0.000	0.000	0.389	0.000	0.000

***Group***	***MAP (mmHg)***			***LVWT (mm)***			***NT-proBNP (ng/L)***		

	***T_0_***	***T_1_***	***T_2_***	***T_0_***	***T_1_***	***T_2_***	***T_0_***	***T_1_***	***T_2_***

Control group ( = 45)	125.32±9.60	123.05±7.10^a^	118.25±7.45^ab^	13.06±0.95	10.25±0.92^a^	9.52±0.90^ab^	8.46±1.02	6.20±0.80^a^	5.70±0.99^ab^
Intervention group ( = 45)	125.76±9.58	119.25±6.90^a^	115.25±4.25^ab^	13.20±0.92	9.85±0.80^a^	8.16±0.74^ab^	8.52±1.09	5.28±1.02^a^	4.46±0.80^ab^
*t*	0.218	2.575	2.346	0.710	2.201	7.830	0.270	4.761	6.535
*P*	0.828	0.012	0.021	0.479	0.030	0.000	0.788	0.000	0.000

***Notes:*** Compared in the same group at T_0_,

a p< 0.05; compared in the same group at T_1_, ^b^p< 0.05.

At T_0_, the levels of inflammatory cytokines in the two groups were lower than those before intervention (*p<*0.05). In the intervention group, IL-6, IL-8 and CRP levels were lower compared with those in the control group at T_1_ and T_2_, with statistically significant differences (*p<*0.05). Table-IV.

**Table-IV:** Comparison of inflammatory cytokines between the two groups (*χ̅*±*S*).

Group	IL-6 (ng/L)			IL-8 (ng/L)			CRP (mg/L)		

	T_0_	T_1_	T_2_	T_0_	T_1_	T_2_	T_0_	T_1_	T_2_
Control group (n = 45)	80.85±12.41	70.34±9.25^a^	59.25±7.85^ab^	84.16±8.46	67.44±5.90^a^	42.25±3.52^ab^	12.15±1.46	10.20±1.21^a^	8.10±0.98^ab^
Intervention group (n = 45)	80.02±12.52	60.30±8.25^a^	45.26±5.25^ab^	84.50±8.29	56.25±4.56^a^	30.25±2.50^ab^	12.27±1.50	8.24±1.02^a^	6.05±0.85^ab^
*t*	0.316	5.434	9.938	0.193	10.067	18.645	0.385	8.308	10.601
*P*	0.753	0.000	0.000	0.848	0.000	0.000	0.701	0.000	0.000

***Notes:*** Compared in the same group at T_0_,

a p< 0.05; compared in the same group at T_1_, ^b^p< 0.05.

## DISCUSSION

Maintaining hemodialysis treatment is a lengthy process that not only causes physiological and psychological pain to patients, but also affects their quality of life and survival rate. Good treatment compliance improves patient prognosis.^10^ Research has shown that the thinking period and thinking period before staged treatment intervention can establish trust between patients and medical staff,^11^,^12^ improve patient cooperation, and the health education during the behavioral period can further enhance patient cooperation, and enable adherence to medical and healthy behaviors to continue, thereby improving treatment effectiveness and improving patient anemia, infection, heart function, and other effects. The results of this study showed that the compliance of patients undergoing staged nursing intervention at 6 and 12 months (71.11%, 91.11%) was higher than that of the conventional nursing intervention group (48.89%, 75.56%) ((*p<*0.05), indicating that staged nursing intervention can improve the compliance of hemodialysis patients, which is consistent with the results of multiple clinical studies.^13^,^14^ The reason is that phased nursing interventions can help patients establish scientific and reasonable rehabilitation goals and plans, enhance their trust in medical staff, weaken their fear, guide them to cope with the disease with a good mentality, and better cooperate with treatment. Research shows that most patients undergoing long-term hemodialysis experience low mood and malnutrition,^15^ which can lead to severe complications such as malnutrition and infection. A good psychological and nutritional balance can reduce the occurrence of severe complications.^16^ The results of this study also showed that the levels of hemoglobin, hematocrit, serum iron, and transferrin saturation in patients receiving staged nursing intervention were higher than those in the conventional nursing group, while the levels of serum total iron synthesis, serum ferritin, IL-6, IL-8, and CRP were lower than those in the conventional nursing group ((*p<*0.05), indicating that staged nursing intervention can effectively reduce the risk of malnutrition and infection in hemodialysis patients.

Relevant literature shows that if a large amount of water in the patient’s body cannot be expelled in a timely and normal manner,^17^,^18^ it can increase the burden on the patient’s heart, and in severe cases, psychological failure may occur; During the process of hemodialysis, using arteriovenous fistula as a dialysis pathway can also increase the return blood flow and exacerbate cardiac negativity;^19^ If anemia occurs in hemodialysis patients, it can cause an increase in heart rate, cardiac output, and cardiac load, leading to complications such as decreased heart function and myocardial ischemia.^20^ The results of this study also showed that the LVEF of patients undergoing staged nursing intervention was higher than that of the conventional nursing group, while LVEDD, LVESD, LVWT, MAP, and NT proBNP were lower than those of the conventional nursing group ((*p<*0.05), indicating that staged nursing intervention can improve cardiac function during hemodialysis in patients. The reason is that phased nursing interventions can promote patients to form good health behavior habits, regularly provide feedback and summary of nursing results, timely solve adverse events during dialysis, ensure the effective and safe implementation of hemodialysis treatment, reduce the probability of heart failure, and effectively improve patients’ cardiac function.

### Limitations:

It includes a small number of patients and no long-term follow-up was conducted. In future studies large number of patients with longer follow up should be performed to study the efficacy of phased therapeutic intervention in the nursing of other chronic diseases.

## CONCLUSIONS

Phased therapeutic intervention can significantly enhance the compliance of new HD patients, as well as further improve their anemia and cardiac function, and reduce inflammatory responses. Therefore, it is worthy of clinical application.

### Authors’ Contributions:

**JS and FG** carried out the studies, data collection and drafted the manuscript.

**DW:** Collected the data and performed the analysis, critical review.

**YW:** Carried out statistical analysis and editing of manuscript. All authors have read,approved the final version and are responsible and accountable for the accuracy for the integrity of the study.
